# Crystal structures of 1-(4-chloro­phen­yl)-4-(4-methyl­phen­yl)-2,5-dioxo-1,2,5,6,7,8-hexa­hydro­quinoline-3-carb­oxy­lic acid and 4-(4-meth­oxy­phen­yl)-1-(4-methyl­phen­yl)-2,5-dioxo-1,2,5,6,7,8-hexa­hydro­quinoline-3-carbo­nitrile

**DOI:** 10.1107/S2056989021002140

**Published:** 2021-03-05

**Authors:** Omyma A. Abd Allah, Manpreet Kaur, Mehmet Akkurt, Shaaban K. Mohamed, Asmaa H. A. Tamam, Sahar M. I. Elgarhy, Jerry P. Jasinski

**Affiliations:** a Chemistry Department, Faculty of Science, Sohag University, 82524 Sohag, Egypt; b Department of Chemistry, Keene State College, 229 Main Street, Keene, NH 03435-2001, USA; c Department of Physics, Faculty of Sciences, Erciyes University, 38039 Kayseri, Turkey; d Chemistry and Environmental Division, Manchester Metropolitan University, Manchester M1 5GD, England; e Chemistry Department, Faculty of Science, Minia University, 61519 El-Minia, Egypt; f Faculty of Science, Department of Bio Chemistry, Beni Suef University, Beni Suef, Egypt

**Keywords:** crystal structure, cyclo­hexene ring, di­hydro­pyridine ring, hexa­hydro­quinoline ring, dimer

## Abstract

In the title compounds C_23_H_21_ClN_2_O_3_ [**I**, namely 1-(4-chloro­phen­yl)-4-(4-methyl­phen­yl)-3,8-dioxo-1,2,5,6,7,8-hexa­hydro­quine-3-carb­oxy­lic acid] and C_24_H_22_N_2_O_3_ [**II**, namely 4-(4-meth­oxy­phen­yl)-1-(4-methyl­phen­yl)-2,5-dioxo-1,2,5,6,7,8-hexa­hydro­quinoline-3-carbo­nitrile], each of the cyclo­hexene and di­hydro­pyridine rings of the 1,2,5,6,7,8-hexa­hydro­quinoline moieties adopts a twisted-boat conformation. The asymmetric units of both compounds **I** and **II** consist of two independent mol­ecules (*A* and *B*). In **II** *A*, three carbon atoms of the cyclo­hexene ring are disordered over two sets of sites in a 0.670 (11):0.330 (11) occupancy ratio. In the crystal of **I**, mol­ecules are linked through classical N—H⋯O hydrogen bonds, forming inversion dimers with an *R* ^2^_2_(8) ring motif and with their mol­ecular planes parallel to the crystallographic (020) plane. Non-classical C—H⋯O hydrogen-bonding inter­actions connect the dimers, resulting in a three-dimensional network. In the crystal of **II**, mol­ecules are linked by C—H⋯N, C—H⋯O and C—H⋯π inter­actions, forming a three-dimensional network.

## Abstract 2

The asymmetric units of compounds **I** and **II** both consist of two independent mol­ecules. In the crystal of **I**, mol­ecules are linked through N—H⋯O hydrogen bonds, forming inversion dimers with 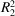
(8) ring motifs and with their mol­ecular planes parallel to (020). C—H⋯O inter­actions connect the dimers, forming a three-dimensional network. In the crystal of **II**, mol­ecules are linked by C—H⋯N, C—H⋯O and C—H⋯π inter­actions, resulting in a three-dimensional network.

## Chemical context   

Quinoline and its derivatives have for some time attracted the attention of both synthetic and biological chemists as a result of their diverse chemical and pharmacological properties (Kumar *et al.*, 2009 ▸). There are a number of natural products bearing the quinoline skeleton that are used as a medicine or employed as lead mol­ecule for the development of new and potent therapeutics (Venkat Reddy *et al.*, 2009 ▸). Quinoline derivatives fused with various heterocycles have already demonstrated potent anti­cancer activity (Afzal *et al.*, 2015 ▸). In addition, it has been found that various quinoline compounds show anti-tuberculosis (TB) activity (Muscia *et al.*, 2014 ▸), anti-inflammatory activity (Psomas & Kessissoglou, 2013 ▸), anti-convulsant effects (Guo *et al.*, 2009 ▸), and anti-malarial parasite effects (Abdel-Gawad *et al.*, 2005 ▸). Furthermore, quinolones have been proved to be very effective in many anti­microbial and anti­oxidant investigations (Praveen *et al.*, 2010 ▸). In this context, we report herein the crystal structures of two derivatives of hexa­hydro­quinoline.
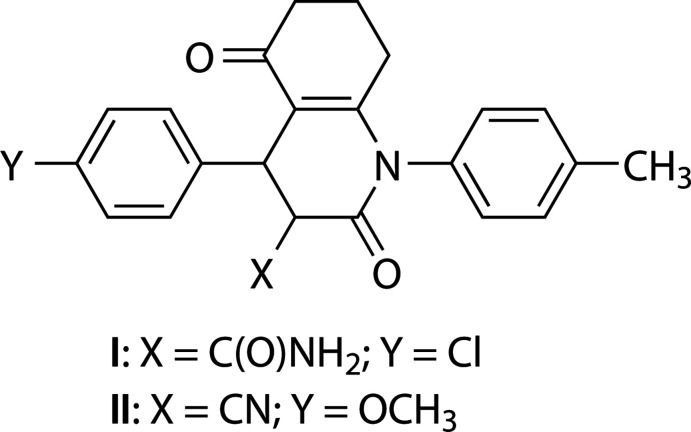


## Structural commentary   

**Figure 1:**
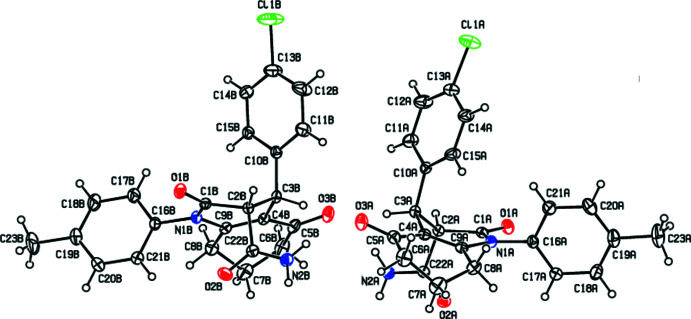
The mol­ecular structure of **I** with the atom-numbering scheme and with displacement ellipsoids drawn at the 30% probability level.

Compound **I** crystallizes in the ortho­rhom­bic space group *Pbca* with *Z* = 16, while compound **II** crystallizes in the monoclinic space group *P*2_1_/*c* with *Z* = 8. The asymmetric units of both compounds (**I** and **II**) each comprise two mol­ecules (*A* and *B*). As shown in Figs. 1 ▸ and 2 ▸, the cyclo­hexene (C4–C9) and di­hydro­pyridine (N1/C1–C4/C9) rings of the 1,2,5,6,7,8-hexa­hydro­quinoline moieties (N1/C1–C9) each adopt a twisted-boat conformation. The puckering parameters (Cremer & Pople, 1975 ▸) of the cyclo­hexene rings are *Q*_T_ = 0.441 (3) Å, θ = 123.3 (3)°, and φ = 1.3 (3)° for **I** *A*, *Q*_T_ = 0.450 (2) Å, θ = 122.0 (3)°, and φ = 4.3 (3)° for **I** *B*, *Q*_T_ = 0.352 (8) Å, θ = 60.7 (11)°, and φ = 188.1 (13)° for **II** *A* (major component of the disorder), and *Q*_T_ = 0.446 (2) Å, θ = 123.5 (3)°, and φ = 355.2 (3)° for **II** *B*. The puckering parameters of the di­hydro­pyridine rings are *Q*_T_ = 0.4929 (18) Å, θ = 64.2 (2)°, and φ = 150.6 (2)° for **I** *A*, *Q*_T_ = 0.4529 (18) Å, θ = 61.1 (2)°, and φ = 139.9 (3)° for **I** *B*, *Q*_T_ = 0.422 (2) Å, θ = 58.9 (3)°, and φ = 138.4 (3)° for **II** *A* and *Q*_T_ = 0.437 (2) Å, θ = 62.5 (3)°, and φ = 142.1 (3)° for **II** *B*.

## Supra­molecular features   

**Figure 3:**
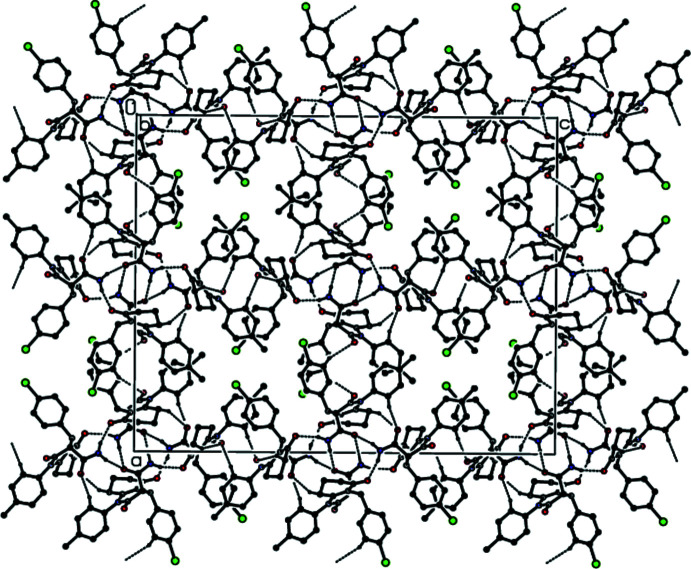
The crystal packing of **I** viewed down the *b* axis showing inter­molecular hydrogen bonds as dashed lines.

**Figure 4:**
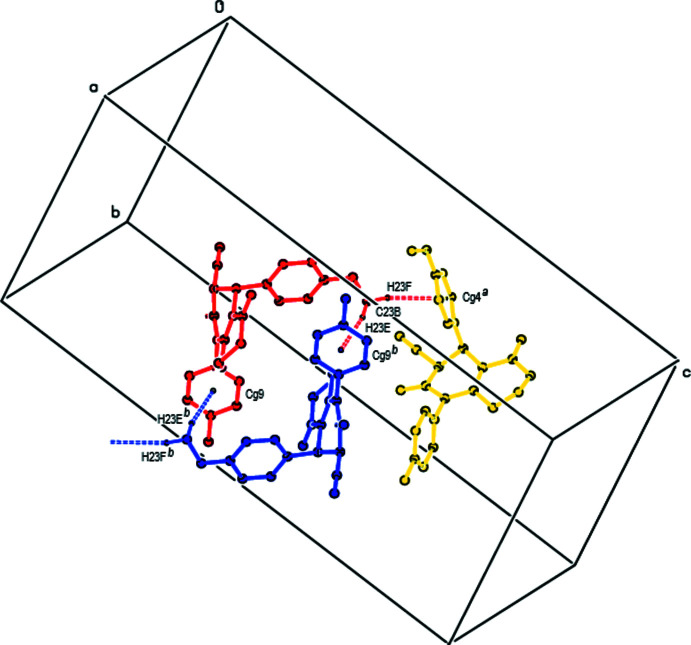
A view of the C—H⋯π inter­actions in **II** shown as dashed lines. [Symmetry codes: (*a*) 1 − *x*, 1 − *y*, 1 − *z*; (*b*) 2 − *x*, 1 − *y*, 1 − *z*].

In the crystal of **I**, two mol­ecules are linked by a pair of inter­molecular N—H⋯O hydrogen bonds with an 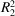
(8) ring motif (Bernstein *et al.*, 1995 ▸; Table 1 ▸), forming an inversion dimer (Fig. 3 ▸). These assemble into a three-dimensional network *via* C—H⋯O inter­actions. In the crystal of **II**, mol­ecules are linked by non-classical C—H⋯O, C—H⋯N and C—H⋯π inter­actions, resulting in a three-dimensional network (Table 2 ▸ and Figs. 4 ▸ and 5 ▸). In the crystals of the two compounds (**I** and **II**) π-π-stacking inter­actions are not observed despite the presence of two aromatic rings in every mol­ecule.

## Database survey   

A search of the Cambridge Structural Database (CSD, Version 5.40, February 2019; Groom *et al.*, 2016 ▸) for the 4-phenyl-2,3,4,6,7,8-hexa­hydro­quinolin-5(1*H*)-one moiety resulted in six closely related hits, *viz.* 2-amino-4-(4-chloro­phen­yl)-1-(4-methyl­phen­yl)-5-oxo-1,4,5,6,7,8-hexa­hydro­quin­oline-3-carbo­nitrile (HUYVUU; Mohamed *et al.*, 2015 ▸), methyl-2,7,7-trimethyl-4-(3-nitro­phen­yl)-5-oxo-1,4,5,6,7,8-hexa­hydro­quinoline-3-carboxyl­ate (TEJQII; Morales *et al.*, 1996 ▸), 3-acetyl-2,7,7-trimethyl-4-phenyl-1,4,5,6,7,8-hexa­hydro-5-quinolone (TEJQOO; Morales *et al.*, 1996 ▸), 4-(4-chloro­phen­yl)-8-methyl-2-oxo-1,2,5,6,7,8-hexa­hydro­quinoline-3-carbo­nitrile (AZOWAO; Asiri *et al.*, 2011 ▸), 8-methyl-2-oxo-4-(thio­phen-2-yl)-1,2,5,6,7,8-hexa­hydro­quinoline-3-carbo­nitrile (XECCAL; Asiri *et al.*, 2012 ▸) and ethyl-2,7,7-trimethyl-5-oxo-4-phenyl-1,4,5,6,7,8-hexa­hydro­quinoline-3-carboxyl­ate (XAYVEA; Kurbanova *et al.*, 2012 ▸).

In compounds **I** and **II**, the observed bond lengths and bond angles are in good agreement with the reported experimental values as found in the structures of HUYVUU, TEJQII, TEJQOO, AZOWAO, XECCAL and XAYVEA. The metrical parameters are, hence, unremarkable.

The angles between the planes of the two benzene rings in **I** and **II** are 52.64 (11)° for **I** *A*, 33.78 (12)° for **I** *B*, 21.80 (11)° for **II** *A* and 19.39 (11)° for **II** *B*, respectively. These angles are notably distinct, even for the two independent mol­ecules in each structure. They are all also significantly larger than the value of 11.52 (7)° found in HUYVUU (the only other example with two benzene rings amongst the related structures). Inter­molecular inter­actions can be weaker or stronger based on the presence or absence or difference of functional groups and the mol­ecular environment, depending on the crystal system, which all affect the mol­ecular conformation. The observed difference in the angles between the two benzene rings may be attributed to these factors.

## Synthesis and crystallization   

***1-(4-Chloro­phen­yl)-4-(4-methyl­phen­yl)-3,8-dioxo-1,2,5,6,7,8-hexa­hydro­quine-3-carboxlic acid***, **I**

A solution of 2-amino-4-(4-chloro­phen­yl)-1-(4-methyl­phen­yl)-5-oxo-1,4,5,6,7,8-hexa­hydro­quinoline-3-carbo­nitrile (2.0 g, 0.0051 mol) in conc. H_2_SO_4_ (20 mL) was stirred for 4 h at room temperature. Then the reaction mixture was poured into ice-cold water. The formed precipitate was collected, filtered off, washed with water and recrystallized from ethanol as pale-yellow crystals; yield 73%, m.p. 518 K.

***4-(4-Meth­oxy­phen­yl)-1-(4-methyl-phen­yl)-2,5-dioxo-1,2,5,6,7,8-hexa­hydro­quinoline-3-carbo­nitrile***, **II**

To a solution of 1,3-cyclohexa­nedione (3.36 g, 0.03 mol) and *p*-toluidine (3.21 g, 0.03 mol) in ethanol (40 ml), a catalytic amount of tri­ethyl­amine was added and the mixture was heated under reflux for 3 h. Ethyl-(2*Z*)-2-cyano-3-(4-meth­oxy­phen­yl)acrylate (6.93 g, 0.03 mol) was added to the reaction mixture while refluxing for another 3 h. The reaction mixture was then cooled to room temperature. The precipitate that formed was filtered off, dried and recrystallized from ethanol solution as orange crystals; yield 67%, m.p. 525 K.

## Refinement   

Crystal data, data collection and structure refinement details are summarized in Table 3 ▸. For **I**, the hydrogen atoms of the NH_2_ group were found in the difference-Fourier map and refined freely. All C-bound H atoms were positioned geometrically (C—H = 0.93–0.98 Å) and refined as riding with *U*_iso_(H) = 1.2*U*_eq_(C) or 1.5*U*_eq_(C-meth­yl). For **II**, all H atoms were positioned geometrically (C—H = 0.95–1.00 Å) and refined as riding with *U*_iso_(H) = 1.2*U*_eq_(C) or 1.5*U*_eq_(C-meth­yl). For **II**, twenty reflections (4 15 10, 3 15 13, 3 14 16, 3 16 7, 3 16 8, 1 3 8, 2 3 0, 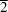 16 3, 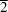 4 10, 2 15 11, 0 14 8, 10 7 13, 1 16 11, 2 1 5, 3 16 4, 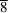 13 7, 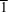 16 4, 0 15 3, 1 16 10, 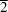 16 11) were omitted as clear outlier data. In **II** *A*, atoms C6*A*, C7*A* and C8*A* of the cyclo­hexane ring are disordered over two sets of sites in a 0.670 (11):0.330 (11) occupancy ratio. The coordinates and the *U* ^ij^ components of the C6*A*, C7*A*, C8*A* and the C6*AA*, C7*AA* and C8*AA* atoms were restrained using SADI and SIMU instructions.

*K* values, which are large only for weak reflections with an *F*_c_/*F*_cmax_ ratio less than 0.005 and less than 0.015 for **I** and **II**, respectively, were observed as 2.713 for **I** and 5.559 for **II**.

## Supplementary Material

Crystal structure: contains datablock(s) I, II, global. DOI: 10.1107/S2056989021002140/yz2005sup1.cif

Structure factors: contains datablock(s) I. DOI: 10.1107/S2056989021002140/yz2005Isup2.hkl

Structure factors: contains datablock(s) II. DOI: 10.1107/S2056989021002140/yz2005IIsup3.hkl

Supporting information file. DOI: 10.1107/S2056989021002140/yz2005Isup4.cml

Supporting information file. DOI: 10.1107/S2056989021002140/yz2005IIsup5.cml

CCDC references: 2064688, 2064687

Additional supporting information:  crystallographic information; 3D view; checkCIF report

## Figures and Tables

**Figure 2:**
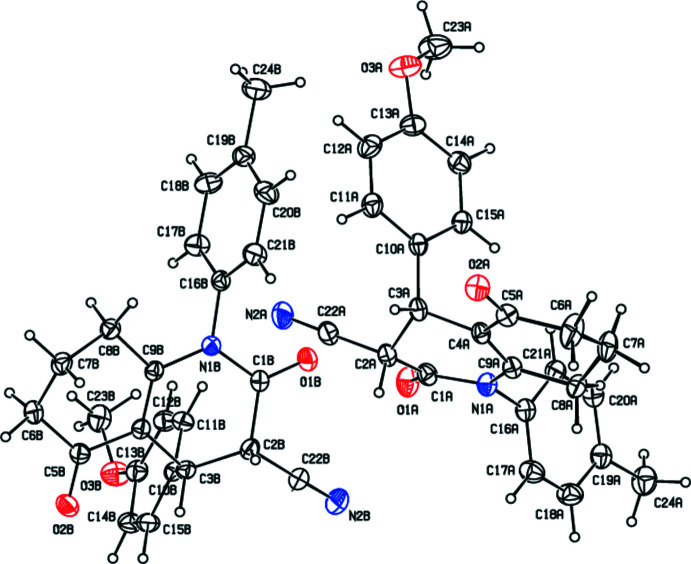
The mol­ecular structure of **II** with the atom-numbering scheme and with displacement ellipsoids drawn at the 20% probability level.

**Figure 5:**
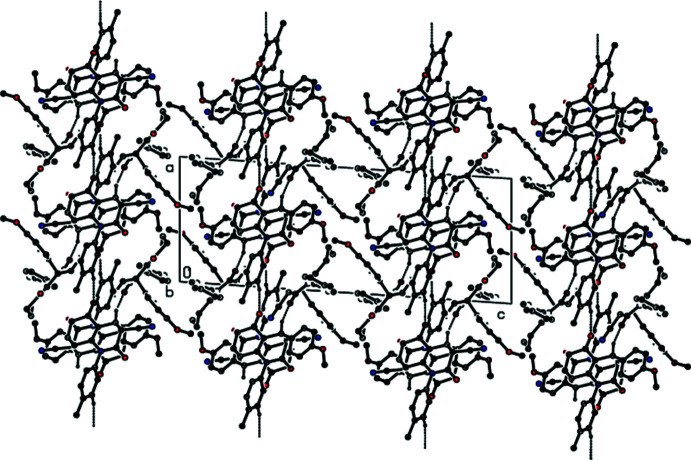
The crystal packing of **II** viewed down the *b* axis showing the inter­molecular hydrogen bonds as dashed lines.

**Table 1:** Hydrogen-bond geometry (Å, °) for **I** [Chem scheme1]

*D*—H⋯*A*	*D*—H	H⋯*A*	*D*⋯*A*	*D*—H⋯*A*
N2*A*—H2*A*2⋯O2*A* ^i^	0.88 (2)	2.12 (3)	2.979 (2)	164 (2)
N2*B*—H2*B*1⋯O2*A* ^ii^	0.86 (2)	2.26 (2)	3.055 (2)	154 (2)
N2*A*—H2*A*1⋯O3*B*	0.86 (2)	2.06 (2)	2.865 (2)	156 (2)
N2*B*—H2*B*2⋯O3*A*	0.85 (3)	2.04 (3)	2.847 (2)	159 (2)
C8*A*—H8*AB*⋯O2*B* ^ii^	0.97	2.50	3.392 (2)	153
C6*B*—H6*BB*⋯O1*B* ^iii^	0.97	2.57	3.430 (2)	148
C14*A*—H14*A*⋯O1*A* ^iv^	0.93	2.54	3.393 (3)	153
C17*B*—H17*B*⋯O1*B* ^v^	0.93	2.52	3.424 (3)	164

Symmetry codes: (i) -x+1, -y, -z+1; (ii) -x+1, -y+1, -z+1; (iii) x, y-1, z; (iv) -x+{\script{3\over 2}}, y+{\script{1\over 2}}, z; (v) -x+1, y-{\script{1\over 2}}, -z+{\script{1\over 2}}.

**Table 2:** Hydrogen-bond geometry (Å, °) for **II** [Chem scheme1] *Cg*4 and *Cg*9 are the centroids of the C10*A*–C15*A* and C16*B*–C21*B* rings, respectively.

*D*—H⋯*A*	*D*—H	H⋯*A*	*D*⋯*A*	*D*—H⋯*A*
C21*A*—H21*A*⋯N2*B* ^i^	0.95	2.65	3.258 (4)	123
C14*B*—H14*B*⋯O3*A* ^ii^	0.95	2.65	3.417 (3)	139
C3*A*—H3*A*⋯O1*B*	1.00	2.37	3.171 (2)	136
C23*B*—H23*E*⋯*Cg*9^iii^	0.98	2.93	3.868 (3)	160
C23*B*—H23*F*⋯*Cg*4^iv^	0.98	2.74	3.710 (3)	169

Symmetry codes: (i) -x+1, y+{\script{1\over 2}}, -z+{\script{1\over 2}}; (ii) x, y-1, z; (iii) -x+2, -y+1, -z+1; (iv) -x+1, -y+1, -z+1.

**Table 3:** Experimental details

	**II**	**II**
Crystal data		
Chemical formula	C_23_H_21_ClN_2_O_3_	C_24_H_22_N_2_O_3_
*M*_r_	408.87	386.43
Crystal system, space group	Orthorhombic, *P* *b* *c* *a*	Monoclinic, *P*2_1_/*c*
Temperature (K)	173	173
*a*, *b*, *c* (Å)	27.9446 (4), 8.4311 (1), 35.0101 (5)	10.3486 (2), 13.9969 (3), 27.5353 (5)
α, β, γ (°)	90, 90, 90	90, 93.797 (2), 90
*V* (Å^3^)	8248.51 (19)	3979.69 (14)
*Z*	16	8
Radiation type	Cu *K*α	Cu *K*α
μ (mm^−1^)	1.86	0.69
Crystal size (mm)	0.16 × 0.10 × 0.05	0.12 × 0.08 × 0.04

Data collection		
Diffractometer	Rigaku Oxford Diffraction Xcalibur, Eos, Gemini	Rigaku Oxford Diffraction Xcalibur, Eos, Gemini
Absorption correction	Multi-scan (*CrysAlis PRO*; Rigaku OD, 2015 ▸)	Multi-scan (*CrysAlis PRO*; Rigaku OD, 2015 ▸)
*T*_min_, *T*_max_	0.614, 1.000	0.945, 1.000
No. of measured, independent and observed [*I* > 2σ(*I*)] reflections	64509, 7983, 6950	15107, 7579, 5380
*R*_int_	0.043	0.027
(sin θ/λ)_max_ (Å^−1^)	0.615	0.615

Refinement		
*R*[*F* ^2^ > 2σ(*F* ^2^)], *wR*(*F* ^2^), *S*	0.050, 0.132, 1.04	0.049, 0.139, 1.04
No. of reflections	7983	7579
No. of parameters	541	555
No. of restraints	0	36
H-atom treatment	H atoms treated by a mixture of independent and constrained refinement	H-atom parameters constrained
Δρ_max_, Δρ_min_ (e Å^−3^)	0.66, −0.55	0.25, −0.16

Computer programs: *CrysAlis PRO* (Rigaku OD, 2015 ▸), *SHELXT* (Sheldrick, 2015*b* ▸), *SHELXL* (Sheldrick, 2015*a* ▸) and *OLEX2* (Dolomanov *et al.*, 2009 ▸).

## supplementary crystallographic information

### 1-(4-Chlorophenyl)-4-(4-methylphenyl)-2,5-dioxo-1,2,5,6,7,8-hexahydroquinoline-3-carboxylic acid (I) Crystal data

**Table:**

C_23_H_21_ClN_2_O_3_	*D*_x_ = 1.317 Mg m^−^^3^
*M**_r_* = 408.87	Cu *K*α radiation, λ = 1.54184 Å
Orthorhombic, *P**b**c**a*	Cell parameters from 21280 reflections
*a* = 27.9446 (4) Å	θ = 4.0–71.5°
*b* = 8.4311 (1) Å	µ = 1.86 mm^−^^1^
*c* = 35.0101 (5) Å	*T* = 173 K
*V* = 8248.51 (19) Å^3^	Prism, pale yellow
*Z* = 16	0.16 × 0.10 × 0.05 mm
*F*(000) = 3424	

### 1-(4-Chlorophenyl)-4-(4-methylphenyl)-2,5-dioxo-1,2,5,6,7,8-hexahydroquinoline-3-carboxylic acid (I) Data collection

**Table:**

Rigaku Oxford Diffraction Xcalibur, Eos, Gemini diffractometer	7983 independent reflections
Radiation source: fine-focus sealed X-ray tube, Enhance (Cu) X-ray Source	6950 reflections with *I* > 2σ(*I*)
Graphite monochromator	*R*_int_ = 0.043
Detector resolution: 16.0416 pixels mm^-1^	θ_max_ = 71.5°, θ_min_ = 4.1°
ω scans	*h* = −29→34
Absorption correction: multi-scan (CrysAlisPro; Rigaku OD, 2015)	*k* = −10→6
*T*_min_ = 0.614, *T*_max_ = 1.000	*l* = −42→42
64509 measured reflections	

### 1-(4-Chlorophenyl)-4-(4-methylphenyl)-2,5-dioxo-1,2,5,6,7,8-hexahydroquinoline-3-carboxylic acid (I) Refinement

**Table:**

Refinement on *F*^2^	Primary atom site location: dual
Least-squares matrix: full	Hydrogen site location: mixed
*R*[*F*^2^ > 2σ(*F*^2^)] = 0.050	H atoms treated by a mixture of independent and constrained refinement
*wR*(*F*^2^) = 0.132	*w* = 1/[σ^2^(*F*_o_^2^) + (0.0636*P*)^2^ + 5.5774*P*] where *P* = (*F*_o_^2^ + 2*F*_c_^2^)/3
*S* = 1.04	(Δ/σ)_max_ < 0.001
7983 reflections	Δρ_max_ = 0.66 e Å^−^^3^
541 parameters	Δρ_min_ = −0.55 e Å^−^^3^
0 restraints	

### 1-(4-Chlorophenyl)-4-(4-methylphenyl)-2,5-dioxo-1,2,5,6,7,8-hexahydroquinoline-3-carboxylic acid (I) Special details

**Table:**

Geometry. All esds (except the esd in the dihedral angle between two l.s. planes) are estimated using the full covariance matrix. The cell esds are taken into account individually in the estimation of esds in distances, angles and torsion angles; correlations between esds in cell parameters are only used when they are defined by crystal symmetry. An approximate (isotropic) treatment of cell esds is used for estimating esds involving l.s. planes.

### 1-(4-Chlorophenyl)-4-(4-methylphenyl)-2,5-dioxo-1,2,5,6,7,8-hexahydroquinoline-3-carboxylic acid (I) Fractional atomic coordinates and isotropic or equivalent isotropic displacement parameters (Å^2^)

**Table:**

	*x*	*y*	*z*	*U*_iso_*/*U*_eq_	
Cl1A	0.82611 (2)	0.31975 (8)	0.40005 (2)	0.0615 (2)	
O1A	0.67114 (5)	0.01077 (15)	0.52217 (4)	0.0322 (3)	
O2A	0.55412 (5)	0.09204 (16)	0.52328 (3)	0.0296 (3)	
O3A	0.58223 (5)	0.60354 (17)	0.44478 (4)	0.0410 (3)	
N1A	0.65291 (5)	0.26677 (17)	0.53641 (4)	0.0243 (3)	
N2A	0.53741 (6)	0.0761 (2)	0.46051 (5)	0.0317 (4)	
C1A	0.64987 (6)	0.1309 (2)	0.51400 (5)	0.0238 (3)	
C2A	0.61843 (6)	0.1434 (2)	0.47878 (5)	0.0226 (3)	
H2A	0.6294	0.0641	0.4603	0.027*	
C3A	0.62358 (6)	0.3088 (2)	0.46017 (5)	0.0240 (3)	
H3AA	0.5998	0.3194	0.4397	0.029*	
C4A	0.61353 (6)	0.4304 (2)	0.49046 (5)	0.0249 (4)	
C5A	0.59223 (7)	0.5801 (2)	0.47852 (6)	0.0312 (4)	
C6A	0.58550 (10)	0.7071 (3)	0.50809 (7)	0.0469 (5)	
H6AA	0.6119	0.7813	0.5065	0.056*	
H6AB	0.5563	0.7649	0.5025	0.056*	
C7A	0.58272 (10)	0.6436 (3)	0.54805 (7)	0.0498 (6)	
H7AA	0.5526	0.5879	0.5513	0.060*	
H7AB	0.5832	0.7316	0.5659	0.060*	
C8A	0.62403 (8)	0.5307 (2)	0.55737 (5)	0.0340 (4)	
H8AA	0.6535	0.5905	0.5596	0.041*	
H8AB	0.6180	0.4790	0.5817	0.041*	
C9A	0.62920 (6)	0.4077 (2)	0.52666 (5)	0.0241 (3)	
C10A	0.67368 (6)	0.3265 (2)	0.44312 (5)	0.0253 (4)	
C11A	0.68482 (8)	0.2534 (3)	0.40888 (6)	0.0398 (5)	
H11A	0.6608	0.2039	0.3949	0.048*	
C12A	0.73127 (9)	0.2530 (3)	0.39512 (7)	0.0476 (6)	
H12A	0.7384	0.2024	0.3722	0.057*	
C13A	0.76677 (7)	0.3277 (2)	0.41543 (6)	0.0368 (4)	
C14A	0.75649 (8)	0.4059 (3)	0.44857 (6)	0.0434 (5)	
H14A	0.7805	0.4579	0.4621	0.052*	
C15A	0.70993 (8)	0.4069 (3)	0.46177 (6)	0.0395 (5)	
H15A	0.7027	0.4632	0.4839	0.047*	
C16A	0.68490 (6)	0.2579 (2)	0.56903 (5)	0.0268 (4)	
C17A	0.67007 (7)	0.1859 (2)	0.60237 (5)	0.0341 (4)	
H17A	0.6388	0.1494	0.6049	0.041*	
C18A	0.70240 (8)	0.1686 (3)	0.63213 (6)	0.0440 (5)	
H18A	0.6923	0.1219	0.6548	0.053*	
C19A	0.74938 (9)	0.2194 (3)	0.62881 (6)	0.0452 (5)	
C20A	0.76314 (8)	0.2944 (3)	0.59536 (7)	0.0448 (5)	
H20A	0.7942	0.3326	0.5930	0.054*	
C21A	0.73132 (7)	0.3134 (3)	0.56536 (6)	0.0375 (4)	
H21A	0.7411	0.3630	0.5429	0.045*	
C22A	0.56659 (6)	0.10306 (19)	0.48956 (5)	0.0228 (3)	
C23A	0.78526 (12)	0.1889 (5)	0.66044 (9)	0.0773 (10)	
H23A	0.7691	0.1890	0.6846	0.116*	
H23B	0.8092	0.2707	0.6602	0.116*	
H23C	0.8002	0.0878	0.6565	0.116*	
Cl1B	0.69954 (3)	0.51675 (14)	0.24096 (2)	0.0960 (4)	
O1B	0.47607 (5)	0.76223 (15)	0.29218 (4)	0.0334 (3)	
O2B	0.43042 (5)	0.68918 (19)	0.37411 (4)	0.0401 (3)	
O3B	0.54926 (5)	0.18004 (16)	0.38319 (4)	0.0376 (3)	
N1B	0.45537 (5)	0.50593 (17)	0.30323 (4)	0.0246 (3)	
N2B	0.49180 (7)	0.7000 (2)	0.41537 (5)	0.0335 (4)	
C1B	0.47972 (6)	0.6448 (2)	0.31185 (5)	0.0240 (3)	
C2B	0.50872 (6)	0.64104 (19)	0.34849 (5)	0.0219 (3)	
H2B	0.5323	0.7270	0.3476	0.026*	
C3B	0.53578 (6)	0.4824 (2)	0.35275 (5)	0.0228 (3)	
H3BA	0.5485	0.4757	0.3788	0.027*	
C4B	0.50099 (6)	0.3490 (2)	0.34697 (5)	0.0238 (3)	
C5B	0.51222 (7)	0.1969 (2)	0.36457 (5)	0.0303 (4)	
C6B	0.47826 (9)	0.0613 (2)	0.35856 (6)	0.0448 (5)	
H6BA	0.4766	−0.0005	0.3819	0.054*	
H6BB	0.4909	−0.0069	0.3387	0.054*	
C7B	0.42824 (9)	0.1114 (3)	0.34759 (6)	0.0432 (5)	
H7BA	0.4126	0.1589	0.3695	0.052*	
H7BB	0.4099	0.0188	0.3401	0.052*	
C8B	0.42911 (7)	0.2300 (2)	0.31484 (6)	0.0345 (4)	
H8BA	0.4389	0.1766	0.2916	0.041*	
H8BB	0.3972	0.2720	0.3109	0.041*	
C9B	0.46301 (6)	0.3640 (2)	0.32309 (5)	0.0247 (3)	
C10B	0.57756 (6)	0.4789 (2)	0.32494 (5)	0.0255 (4)	
C11B	0.62165 (7)	0.5355 (3)	0.33652 (6)	0.0432 (5)	
H11B	0.6260	0.5660	0.3618	0.052*	
C12B	0.65946 (9)	0.5476 (4)	0.31115 (8)	0.0608 (8)	
H12B	0.6890	0.5862	0.3192	0.073*	
C13B	0.65256 (8)	0.5013 (3)	0.27362 (7)	0.0505 (6)	
C14B	0.60972 (8)	0.4409 (3)	0.26148 (6)	0.0376 (4)	
H14B	0.6058	0.4080	0.2363	0.045*	
C15B	0.57242 (7)	0.4297 (2)	0.28716 (5)	0.0296 (4)	
H15B	0.5433	0.3884	0.2791	0.035*	
C16B	0.41761 (6)	0.5176 (2)	0.27496 (5)	0.0280 (4)	
C17B	0.42624 (8)	0.4736 (3)	0.23774 (6)	0.0410 (5)	
H17B	0.4561	0.4357	0.2305	0.049*	
C18B	0.38942 (9)	0.4867 (4)	0.21121 (6)	0.0557 (7)	
H18B	0.3950	0.4580	0.1859	0.067*	
C19B	0.34492 (9)	0.5414 (4)	0.22167 (7)	0.0554 (7)	
C20B	0.33726 (7)	0.5846 (3)	0.25939 (6)	0.0449 (5)	
H20B	0.3073	0.6209	0.2668	0.054*	
C21B	0.37358 (7)	0.5744 (3)	0.28612 (6)	0.0344 (4)	
H21B	0.3683	0.6055	0.3113	0.041*	
C22B	0.47312 (6)	0.6765 (2)	0.38092 (5)	0.0262 (4)	
C23B	0.30543 (12)	0.5582 (7)	0.19237 (10)	0.1053 (16)	
H23D	0.2808	0.4815	0.1975	0.158*	
H23E	0.3183	0.5407	0.1673	0.158*	
H23F	0.2921	0.6630	0.1938	0.158*	
H2A2	0.5079 (9)	0.045 (3)	0.4652 (6)	0.031 (6)*	
H2B1	0.4729 (9)	0.732 (3)	0.4330 (7)	0.037 (6)*	
H2A1	0.5470 (9)	0.085 (3)	0.4374 (7)	0.038 (6)*	
H2B2	0.5214 (10)	0.689 (3)	0.4200 (7)	0.043 (7)*	

### 1-(4-Chlorophenyl)-4-(4-methylphenyl)-2,5-dioxo-1,2,5,6,7,8-hexahydroquinoline-3-carboxylic acid (I) Atomic displacement parameters (Å^2^)

**Table:**

	*U*^11^	*U*^22^	*U*^33^	*U*^12^	*U*^13^	*U*^23^
Cl1A	0.0364 (3)	0.0663 (4)	0.0816 (5)	−0.0009 (3)	0.0225 (3)	−0.0174 (3)
O1A	0.0331 (7)	0.0261 (7)	0.0375 (7)	0.0074 (5)	−0.0058 (5)	−0.0021 (5)
O2A	0.0288 (6)	0.0351 (7)	0.0251 (6)	−0.0052 (5)	0.0032 (5)	−0.0055 (5)
O3A	0.0462 (8)	0.0361 (8)	0.0406 (8)	0.0084 (6)	−0.0093 (6)	0.0078 (6)
N1A	0.0246 (7)	0.0238 (7)	0.0246 (7)	0.0008 (6)	−0.0018 (6)	−0.0010 (6)
N2A	0.0252 (8)	0.0441 (10)	0.0259 (8)	−0.0092 (7)	−0.0025 (6)	0.0044 (7)
C1A	0.0209 (8)	0.0230 (8)	0.0276 (8)	−0.0008 (7)	0.0020 (6)	0.0006 (7)
C2A	0.0230 (8)	0.0207 (8)	0.0240 (8)	0.0002 (6)	0.0005 (6)	−0.0021 (6)
C3A	0.0257 (8)	0.0230 (8)	0.0233 (8)	−0.0006 (7)	−0.0023 (6)	0.0007 (6)
C4A	0.0243 (8)	0.0215 (8)	0.0288 (9)	−0.0019 (7)	0.0021 (7)	0.0003 (7)
C5A	0.0297 (9)	0.0249 (9)	0.0391 (10)	0.0003 (7)	−0.0009 (8)	0.0030 (8)
C6A	0.0615 (15)	0.0252 (10)	0.0542 (13)	0.0112 (10)	−0.0046 (11)	−0.0017 (9)
C7A	0.0705 (16)	0.0353 (11)	0.0437 (12)	0.0156 (11)	0.0062 (11)	−0.0097 (9)
C8A	0.0460 (11)	0.0265 (9)	0.0294 (9)	−0.0005 (8)	0.0033 (8)	−0.0046 (7)
C9A	0.0236 (8)	0.0205 (8)	0.0283 (8)	−0.0028 (6)	0.0028 (7)	−0.0003 (7)
C10A	0.0281 (9)	0.0250 (8)	0.0229 (8)	−0.0013 (7)	0.0002 (7)	0.0032 (6)
C11A	0.0408 (11)	0.0482 (12)	0.0305 (10)	−0.0114 (10)	0.0021 (8)	−0.0116 (9)
C12A	0.0474 (13)	0.0529 (13)	0.0423 (12)	−0.0075 (11)	0.0142 (10)	−0.0205 (10)
C13A	0.0316 (10)	0.0366 (10)	0.0423 (11)	−0.0015 (8)	0.0097 (8)	−0.0003 (8)
C14A	0.0335 (11)	0.0631 (14)	0.0336 (10)	−0.0162 (10)	0.0034 (8)	−0.0070 (10)
C15A	0.0371 (11)	0.0538 (13)	0.0275 (9)	−0.0129 (9)	0.0060 (8)	−0.0105 (9)
C16A	0.0271 (9)	0.0274 (9)	0.0257 (8)	0.0025 (7)	−0.0035 (7)	−0.0027 (7)
C17A	0.0283 (9)	0.0425 (11)	0.0314 (9)	0.0042 (8)	0.0018 (7)	0.0034 (8)
C18A	0.0493 (12)	0.0545 (13)	0.0282 (10)	0.0092 (10)	−0.0039 (9)	0.0052 (9)
C19A	0.0477 (12)	0.0477 (13)	0.0402 (11)	0.0084 (10)	−0.0178 (10)	−0.0065 (10)
C20A	0.0309 (10)	0.0487 (13)	0.0548 (13)	−0.0066 (9)	−0.0126 (9)	−0.0066 (10)
C21A	0.0344 (10)	0.0422 (11)	0.0360 (10)	−0.0079 (9)	−0.0036 (8)	0.0021 (9)
C22A	0.0242 (8)	0.0162 (7)	0.0279 (8)	0.0010 (6)	0.0011 (7)	−0.0010 (6)
C23A	0.0704 (19)	0.097 (2)	0.0647 (18)	0.0068 (17)	−0.0403 (16)	0.0008 (17)
Cl1B	0.0615 (4)	0.1578 (9)	0.0687 (5)	−0.0455 (5)	0.0382 (4)	−0.0341 (5)
O1B	0.0397 (7)	0.0276 (7)	0.0327 (7)	−0.0015 (6)	−0.0062 (6)	0.0098 (5)
O2B	0.0275 (7)	0.0539 (9)	0.0388 (7)	0.0046 (6)	0.0030 (6)	−0.0066 (7)
O3B	0.0484 (8)	0.0321 (7)	0.0322 (7)	0.0089 (6)	−0.0075 (6)	0.0075 (6)
N1B	0.0254 (7)	0.0244 (7)	0.0241 (7)	−0.0002 (6)	−0.0039 (6)	0.0020 (6)
N2B	0.0365 (9)	0.0389 (9)	0.0251 (8)	0.0098 (7)	0.0016 (7)	−0.0039 (7)
C1B	0.0247 (8)	0.0236 (8)	0.0238 (8)	0.0017 (7)	0.0013 (6)	0.0018 (7)
C2B	0.0234 (8)	0.0186 (8)	0.0236 (8)	0.0000 (6)	−0.0015 (6)	0.0001 (6)
C3B	0.0274 (8)	0.0218 (8)	0.0192 (7)	0.0037 (7)	−0.0041 (6)	−0.0011 (6)
C4B	0.0306 (9)	0.0204 (8)	0.0204 (8)	0.0023 (7)	0.0010 (6)	0.0000 (6)
C5B	0.0455 (11)	0.0231 (9)	0.0222 (8)	0.0049 (8)	0.0009 (8)	0.0004 (7)
C6B	0.0723 (16)	0.0214 (9)	0.0407 (11)	−0.0050 (10)	−0.0097 (11)	0.0064 (8)
C7B	0.0568 (14)	0.0321 (10)	0.0406 (11)	−0.0195 (10)	−0.0031 (10)	0.0044 (9)
C8B	0.0390 (10)	0.0299 (10)	0.0347 (10)	−0.0085 (8)	−0.0055 (8)	0.0011 (8)
C9B	0.0301 (9)	0.0219 (8)	0.0220 (8)	0.0003 (7)	0.0022 (7)	0.0001 (6)
C10B	0.0260 (8)	0.0245 (8)	0.0258 (8)	0.0036 (7)	−0.0020 (7)	−0.0021 (7)
C11B	0.0329 (10)	0.0629 (14)	0.0338 (10)	−0.0063 (10)	−0.0015 (8)	−0.0183 (10)
C12B	0.0325 (11)	0.093 (2)	0.0563 (15)	−0.0212 (13)	0.0061 (10)	−0.0279 (14)
C13B	0.0378 (12)	0.0696 (16)	0.0441 (12)	−0.0095 (11)	0.0146 (10)	−0.0107 (11)
C14B	0.0399 (11)	0.0463 (11)	0.0267 (9)	0.0032 (9)	0.0028 (8)	−0.0048 (8)
C15B	0.0283 (9)	0.0348 (10)	0.0256 (9)	0.0016 (8)	−0.0032 (7)	−0.0033 (7)
C16B	0.0280 (9)	0.0304 (9)	0.0257 (9)	−0.0013 (7)	−0.0049 (7)	0.0034 (7)
C17B	0.0364 (11)	0.0569 (13)	0.0296 (10)	0.0075 (10)	−0.0037 (8)	−0.0046 (9)
C18B	0.0534 (14)	0.0867 (19)	0.0269 (10)	0.0080 (13)	−0.0111 (10)	−0.0080 (11)
C19B	0.0399 (12)	0.089 (2)	0.0375 (12)	0.0031 (13)	−0.0149 (10)	0.0056 (12)
C20B	0.0261 (10)	0.0658 (15)	0.0429 (12)	0.0036 (10)	−0.0041 (8)	0.0047 (10)
C21B	0.0305 (10)	0.0441 (11)	0.0287 (9)	−0.0003 (8)	−0.0008 (7)	0.0028 (8)
C22B	0.0301 (9)	0.0193 (8)	0.0291 (9)	0.0029 (7)	0.0021 (7)	0.0013 (6)
C23B	0.0598 (19)	0.202 (5)	0.0542 (18)	0.023 (3)	−0.0332 (15)	−0.004 (2)

### 1-(4-Chlorophenyl)-4-(4-methylphenyl)-2,5-dioxo-1,2,5,6,7,8-hexahydroquinoline-3-carboxylic acid (I) Geometric parameters (Å, º)

**Table:**

Cl1A—C13A	1.745 (2)	Cl1B—C13B	1.746 (2)
O1A—C1A	1.209 (2)	O1B—C1B	1.210 (2)
O2A—C22A	1.234 (2)	O2B—C22B	1.221 (2)
O3A—C5A	1.230 (2)	O3B—C5B	1.232 (2)
N1A—C1A	1.391 (2)	N1B—C1B	1.387 (2)
N1A—C9A	1.403 (2)	N1B—C9B	1.400 (2)
N1A—C16A	1.452 (2)	N1B—C16B	1.450 (2)
N2A—C22A	1.323 (2)	N2B—C22B	1.329 (2)
N2A—H2A2	0.88 (2)	N2B—H2B1	0.85 (3)
N2A—H2A1	0.86 (3)	N2B—H2B2	0.85 (3)
C1A—C2A	1.518 (2)	C1B—C2B	1.518 (2)
C2A—C22A	1.535 (2)	C2B—C22B	1.539 (2)
C2A—C3A	1.546 (2)	C2B—C3B	1.544 (2)
C2A—H2A	0.9800	C2B—H2B	0.9800
C3A—C4A	1.502 (2)	C3B—C4B	1.500 (2)
C3A—C10A	1.529 (2)	C3B—C10B	1.521 (2)
C3A—H3AA	0.9800	C3B—H3BA	0.9800
C4A—C9A	1.355 (3)	C4B—C9B	1.357 (2)
C4A—C5A	1.456 (3)	C4B—C5B	1.457 (2)
C5A—C6A	1.501 (3)	C5B—C6B	1.500 (3)
C6A—C7A	1.500 (3)	C6B—C7B	1.510 (3)
C6A—H6AA	0.9700	C6B—H6BA	0.9700
C6A—H6AB	0.9700	C6B—H6BB	0.9700
C7A—C8A	1.532 (3)	C7B—C8B	1.522 (3)
C7A—H7AA	0.9700	C7B—H7BA	0.9700
C7A—H7AB	0.9700	C7B—H7BB	0.9700
C8A—C9A	1.500 (2)	C8B—C9B	1.503 (3)
C8A—H8AA	0.9700	C8B—H8BA	0.9700
C8A—H8AB	0.9700	C8B—H8BB	0.9700
C10A—C15A	1.383 (3)	C10B—C11B	1.382 (3)
C10A—C11A	1.383 (3)	C10B—C15B	1.394 (2)
C11A—C12A	1.385 (3)	C11B—C12B	1.384 (3)
C11A—H11A	0.9300	C11B—H11B	0.9300
C12A—C13A	1.373 (3)	C12B—C13B	1.384 (3)
C12A—H12A	0.9300	C12B—H12B	0.9300
C13A—C14A	1.365 (3)	C13B—C14B	1.369 (3)
C14A—C15A	1.381 (3)	C14B—C15B	1.380 (3)
C14A—H14A	0.9300	C14B—H14B	0.9300
C15A—H15A	0.9300	C15B—H15B	0.9300
C16A—C17A	1.379 (3)	C16B—C17B	1.376 (3)
C16A—C21A	1.385 (3)	C16B—C21B	1.377 (3)
C17A—C18A	1.387 (3)	C17B—C18B	1.391 (3)
C17A—H17A	0.9300	C17B—H17B	0.9300
C18A—C19A	1.386 (4)	C18B—C19B	1.376 (4)
C18A—H18A	0.9300	C18B—H18B	0.9300
C19A—C20A	1.385 (3)	C19B—C20B	1.386 (3)
C19A—C23A	1.516 (3)	C19B—C23B	1.513 (3)
C20A—C21A	1.386 (3)	C20B—C21B	1.383 (3)
C20A—H20A	0.9300	C20B—H20B	0.9300
C21A—H21A	0.9300	C21B—H21B	0.9300
C23A—H23A	0.9600	C23B—H23D	0.9600
C23A—H23B	0.9600	C23B—H23E	0.9600
C23A—H23C	0.9600	C23B—H23F	0.9600
			
C1A—N1A—C9A	122.11 (14)	C1B—N1B—C9B	122.57 (14)
C1A—N1A—C16A	116.03 (14)	C1B—N1B—C16B	116.61 (14)
C9A—N1A—C16A	121.72 (14)	C9B—N1B—C16B	120.53 (14)
C22A—N2A—H2A2	118.9 (14)	C22B—N2B—H2B1	117.2 (16)
C22A—N2A—H2A1	121.2 (16)	C22B—N2B—H2B2	122.7 (17)
H2A2—N2A—H2A1	120 (2)	H2B1—N2B—H2B2	120 (2)
O1A—C1A—N1A	121.78 (16)	O1B—C1B—N1B	121.69 (16)
O1A—C1A—C2A	122.32 (16)	O1B—C1B—C2B	122.89 (16)
N1A—C1A—C2A	115.90 (14)	N1B—C1B—C2B	115.34 (14)
C1A—C2A—C22A	109.34 (14)	C1B—C2B—C22B	105.92 (14)
C1A—C2A—C3A	110.54 (14)	C1B—C2B—C3B	111.19 (13)
C22A—C2A—C3A	113.03 (14)	C22B—C2B—C3B	114.42 (13)
C1A—C2A—H2A	107.9	C1B—C2B—H2B	108.4
C22A—C2A—H2A	107.9	C22B—C2B—H2B	108.4
C3A—C2A—H2A	107.9	C3B—C2B—H2B	108.4
C4A—C3A—C10A	112.35 (14)	C4B—C3B—C10B	113.37 (14)
C4A—C3A—C2A	107.50 (14)	C4B—C3B—C2B	108.59 (14)
C10A—C3A—C2A	109.75 (14)	C10B—C3B—C2B	109.32 (14)
C4A—C3A—H3AA	109.1	C4B—C3B—H3BA	108.5
C10A—C3A—H3AA	109.1	C10B—C3B—H3BA	108.5
C2A—C3A—H3AA	109.1	C2B—C3B—H3BA	108.5
C9A—C4A—C5A	121.53 (16)	C9B—C4B—C5B	120.72 (16)
C9A—C4A—C3A	120.26 (16)	C9B—C4B—C3B	121.35 (15)
C5A—C4A—C3A	117.73 (16)	C5B—C4B—C3B	117.59 (15)
O3A—C5A—C4A	120.51 (18)	O3B—C5B—C4B	120.41 (17)
O3A—C5A—C6A	121.28 (18)	O3B—C5B—C6B	121.21 (17)
C4A—C5A—C6A	118.12 (17)	C4B—C5B—C6B	118.37 (17)
C7A—C6A—C5A	113.28 (18)	C5B—C6B—C7B	114.10 (17)
C7A—C6A—H6AA	108.9	C5B—C6B—H6BA	108.7
C5A—C6A—H6AA	108.9	C7B—C6B—H6BA	108.7
C7A—C6A—H6AB	108.9	C5B—C6B—H6BB	108.7
C5A—C6A—H6AB	108.9	C7B—C6B—H6BB	108.7
H6AA—C6A—H6AB	107.7	H6BA—C6B—H6BB	107.6
C6A—C7A—C8A	112.44 (19)	C6B—C7B—C8B	111.15 (18)
C6A—C7A—H7AA	109.1	C6B—C7B—H7BA	109.4
C8A—C7A—H7AA	109.1	C8B—C7B—H7BA	109.4
C6A—C7A—H7AB	109.1	C6B—C7B—H7BB	109.4
C8A—C7A—H7AB	109.1	C8B—C7B—H7BB	109.4
H7AA—C7A—H7AB	107.8	H7BA—C7B—H7BB	108.0
C9A—C8A—C7A	110.44 (17)	C9B—C8B—C7B	111.05 (16)
C9A—C8A—H8AA	109.6	C9B—C8B—H8BA	109.4
C7A—C8A—H8AA	109.6	C7B—C8B—H8BA	109.4
C9A—C8A—H8AB	109.6	C9B—C8B—H8BB	109.4
C7A—C8A—H8AB	109.6	C7B—C8B—H8BB	109.4
H8AA—C8A—H8AB	108.1	H8BA—C8B—H8BB	108.0
C4A—C9A—N1A	120.00 (15)	C4B—C9B—N1B	120.31 (16)
C4A—C9A—C8A	122.81 (16)	C4B—C9B—C8B	122.79 (16)
N1A—C9A—C8A	117.15 (15)	N1B—C9B—C8B	116.81 (15)
C15A—C10A—C11A	117.57 (17)	C11B—C10B—C15B	118.23 (17)
C15A—C10A—C3A	122.30 (16)	C11B—C10B—C3B	119.34 (16)
C11A—C10A—C3A	120.04 (16)	C15B—C10B—C3B	122.30 (16)
C10A—C11A—C12A	120.90 (19)	C10B—C11B—C12B	121.18 (19)
C10A—C11A—H11A	119.5	C10B—C11B—H11B	119.4
C12A—C11A—H11A	119.5	C12B—C11B—H11B	119.4
C13A—C12A—C11A	119.72 (19)	C11B—C12B—C13B	118.8 (2)
C13A—C12A—H12A	120.1	C11B—C12B—H12B	120.6
C11A—C12A—H12A	120.1	C13B—C12B—H12B	120.6
C14A—C13A—C12A	120.63 (19)	C14B—C13B—C12B	121.4 (2)
C14A—C13A—Cl1A	118.75 (17)	C14B—C13B—Cl1B	118.84 (18)
C12A—C13A—Cl1A	120.60 (16)	C12B—C13B—Cl1B	119.73 (18)
C13A—C14A—C15A	119.05 (19)	C13B—C14B—C15B	118.95 (19)
C13A—C14A—H14A	120.5	C13B—C14B—H14B	120.5
C15A—C14A—H14A	120.5	C15B—C14B—H14B	120.5
C14A—C15A—C10A	121.97 (19)	C14B—C15B—C10B	121.35 (18)
C14A—C15A—H15A	119.0	C14B—C15B—H15B	119.3
C10A—C15A—H15A	119.0	C10B—C15B—H15B	119.3
C17A—C16A—C21A	120.58 (17)	C17B—C16B—C21B	121.26 (18)
C17A—C16A—N1A	120.22 (16)	C17B—C16B—N1B	120.03 (17)
C21A—C16A—N1A	119.08 (16)	C21B—C16B—N1B	118.71 (16)
C16A—C17A—C18A	119.10 (19)	C16B—C17B—C18B	118.8 (2)
C16A—C17A—H17A	120.4	C16B—C17B—H17B	120.6
C18A—C17A—H17A	120.4	C18B—C17B—H17B	120.6
C19A—C18A—C17A	121.4 (2)	C19B—C18B—C17B	121.2 (2)
C19A—C18A—H18A	119.3	C19B—C18B—H18B	119.4
C17A—C18A—H18A	119.3	C17B—C18B—H18B	119.4
C20A—C19A—C18A	118.36 (19)	C18B—C19B—C20B	118.8 (2)
C20A—C19A—C23A	120.7 (2)	C18B—C19B—C23B	120.7 (3)
C18A—C19A—C23A	120.9 (2)	C20B—C19B—C23B	120.6 (3)
C19A—C20A—C21A	121.0 (2)	C21B—C20B—C19B	121.0 (2)
C19A—C20A—H20A	119.5	C21B—C20B—H20B	119.5
C21A—C20A—H20A	119.5	C19B—C20B—H20B	119.5
C16A—C21A—C20A	119.4 (2)	C16B—C21B—C20B	119.03 (19)
C16A—C21A—H21A	120.3	C16B—C21B—H21B	120.5
C20A—C21A—H21A	120.3	C20B—C21B—H21B	120.5
O2A—C22A—N2A	123.25 (16)	O2B—C22B—N2B	123.20 (17)
O2A—C22A—C2A	121.21 (15)	O2B—C22B—C2B	120.30 (16)
N2A—C22A—C2A	115.49 (15)	N2B—C22B—C2B	116.41 (16)
C19A—C23A—H23A	109.5	C19B—C23B—H23D	109.5
C19A—C23A—H23B	109.5	C19B—C23B—H23E	109.5
H23A—C23A—H23B	109.5	H23D—C23B—H23E	109.5
C19A—C23A—H23C	109.5	C19B—C23B—H23F	109.5
H23A—C23A—H23C	109.5	H23D—C23B—H23F	109.5
H23B—C23A—H23C	109.5	H23E—C23B—H23F	109.5

### 1-(4-Chlorophenyl)-4-(4-methylphenyl)-2,5-dioxo-1,2,5,6,7,8-hexahydroquinoline-3-carboxylic acid (I) Hydrogen-bond geometry (Å, º)

**Table:**

*D*—H···*A*	*D*—H	H···*A*	*D*···*A*	*D*—H···*A*
N2*A*—H2*A*2···O2*A*^i^	0.88 (2)	2.12 (3)	2.979 (2)	164 (2)
N2*B*—H2*B*1···O2*A*^ii^	0.86 (2)	2.26 (2)	3.055 (2)	154 (2)
N2*A*—H2*A*1···O3*B*	0.86 (2)	2.06 (2)	2.865 (2)	156 (2)
N2*B*—H2*B*2···O3*A*	0.85 (3)	2.04 (3)	2.847 (2)	159 (2)
C3*A*—H3*AA*···O3*A*	0.98	2.45	2.793 (2)	100
C8*A*—H8*AB*···O2*B*^ii^	0.97	2.50	3.392 (2)	153
C6*B*—H6*BB*···O1*B*^iii^	0.97	2.57	3.430 (2)	148
C14*A*—H14*A*···O1*A*^iv^	0.93	2.54	3.393 (3)	153
C17*A*—H17*A*···O2*B*^ii^	0.93	2.48	3.110 (2)	125
C17*B*—H17*B*···O1*B*^v^	0.93	2.52	3.424 (3)	164

Symmetry codes: (i) −*x*+1, −*y*, −*z*+1; (ii) −*x*+1, −*y*+1, −*z*+1; (iii) *x*, *y*−1, *z*; (iv) −*x*+3/2, *y*+1/2, *z*; (v) −*x*+1, *y*−1/2, −*z*+1/2.

### 4-(4-Methoxyphenyl)-1-(4-methylphenyl)-2,5-dioxo-1,2,5,6,7,8-hexahydroquinoline-3-carbonitrile (II) Crystal data

**Table:**

C_24_H_22_N_2_O_3_	*F*(000) = 1632
*M**_r_* = 386.43	*D*_x_ = 1.290 Mg m^−^^3^
Monoclinic, *P*2_1_/*c*	Cu *K*α radiation, λ = 1.54184 Å
*a* = 10.3486 (2) Å	Cell parameters from 3849 reflections
*b* = 13.9969 (3) Å	θ = 4.3–70.8°
*c* = 27.5353 (5) Å	µ = 0.69 mm^−^^1^
β = 93.797 (2)°	*T* = 173 K
*V* = 3979.69 (14) Å^3^	Irregular, orange
*Z* = 8	0.12 × 0.08 × 0.04 mm

### 4-(4-Methoxyphenyl)-1-(4-methylphenyl)-2,5-dioxo-1,2,5,6,7,8-hexahydroquinoline-3-carbonitrile (II) Data collection

**Table:**

Rigaku Oxford Diffraction Xcalibur, Eos, Gemini diffractometer	5380 reflections with *I* > 2σ(*I*)
Detector resolution: 16.0416 pixels mm^-1^	*R*_int_ = 0.027
ω scans	θ_max_ = 71.4°, θ_min_ = 3.5°
Absorption correction: multi-scan (CrysAlisPro; Rigaku OD, 2015)	*h* = −9→12
*T*_min_ = 0.945, *T*_max_ = 1.000	*k* = −10→16
15107 measured reflections	*l* = −26→33
7579 independent reflections	

### 4-(4-Methoxyphenyl)-1-(4-methylphenyl)-2,5-dioxo-1,2,5,6,7,8-hexahydroquinoline-3-carbonitrile (II) Refinement

**Table:**

Refinement on *F*^2^	Primary atom site location: dual
Least-squares matrix: full	Hydrogen site location: inferred from neighbouring sites
*R*[*F*^2^ > 2σ(*F*^2^)] = 0.049	H-atom parameters constrained
*wR*(*F*^2^) = 0.139	*w* = 1/[σ^2^(*F*_o_^2^) + (0.0628*P*)^2^ + 0.1965*P*] where *P* = (*F*_o_^2^ + 2*F*_c_^2^)/3
*S* = 1.03	(Δ/σ)_max_ < 0.001
7579 reflections	Δρ_max_ = 0.25 e Å^−^^3^
555 parameters	Δρ_min_ = −0.16 e Å^−^^3^
36 restraints	

### 4-(4-Methoxyphenyl)-1-(4-methylphenyl)-2,5-dioxo-1,2,5,6,7,8-hexahydroquinoline-3-carbonitrile (II) Special details

**Table:**

Geometry. All esds (except the esd in the dihedral angle between two l.s. planes) are estimated using the full covariance matrix. The cell esds are taken into account individually in the estimation of esds in distances, angles and torsion angles; correlations between esds in cell parameters are only used when they are defined by crystal symmetry. An approximate (isotropic) treatment of cell esds is used for estimating esds involving l.s. planes.

### 4-(4-Methoxyphenyl)-1-(4-methylphenyl)-2,5-dioxo-1,2,5,6,7,8-hexahydroquinoline-3-carbonitrile (II) Fractional atomic coordinates and isotropic or equivalent isotropic displacement parameters (Å^2^)

**Table:**

	*x*	*y*	*z*	*U*_iso_*/*U*_eq_	Occ. (<1)
O1A	0.36261 (17)	0.53971 (14)	0.33466 (6)	0.0704 (5)	
O2A	0.78343 (15)	0.78301 (13)	0.24000 (6)	0.0635 (4)	
O3A	0.5360 (2)	0.99458 (16)	0.43140 (8)	0.0866 (6)	
N1A	0.40024 (16)	0.60665 (14)	0.26190 (6)	0.0490 (4)	
N2A	0.6257 (3)	0.56408 (18)	0.41665 (8)	0.0773 (6)	
C1A	0.4369 (2)	0.57641 (15)	0.30847 (7)	0.0502 (5)	
C2A	0.5814 (2)	0.58943 (16)	0.32306 (7)	0.0499 (5)	
H2A	0.628554	0.537522	0.306612	0.060*	
C3A	0.63700 (18)	0.68516 (15)	0.30610 (7)	0.0462 (4)	
H3A	0.733419	0.678557	0.307960	0.055*	
C4A	0.59327 (19)	0.69818 (15)	0.25308 (7)	0.0440 (4)	
C5A	0.6773 (2)	0.75536 (16)	0.22368 (8)	0.0509 (5)	
C6A	0.6240 (7)	0.7794 (6)	0.1737 (2)	0.093 (3)	0.670 (11)
H6A1	0.647523	0.846564	0.167295	0.112*	0.670 (11)
H6A2	0.669856	0.739274	0.150722	0.112*	0.670 (11)
C7A	0.4928 (5)	0.7699 (5)	0.16172 (18)	0.0727 (19)	0.670 (11)
H7A1	0.478234	0.768970	0.125825	0.087*	0.670 (11)
H7A2	0.447588	0.826567	0.173844	0.087*	0.670 (11)
C8A	0.4343 (10)	0.6823 (10)	0.1818 (3)	0.058 (3)	0.670 (11)
H8A1	0.454564	0.627035	0.161223	0.070*	0.670 (11)
H8A2	0.338993	0.689814	0.180160	0.070*	0.670 (11)
C6AA	0.6417 (13)	0.7776 (12)	0.1706 (4)	0.081 (6)	0.330 (11)
H6A3	0.609006	0.843920	0.167569	0.097*	0.330 (11)
H6A4	0.719692	0.772369	0.151825	0.097*	0.330 (11)
C7AA	0.5411 (9)	0.7106 (9)	0.1504 (2)	0.062 (3)	0.330 (11)
H7A3	0.587817	0.655632	0.137269	0.074*	0.330 (11)
H7A4	0.496696	0.743252	0.122131	0.074*	0.330 (11)
C8AA	0.4327 (18)	0.668 (2)	0.1804 (5)	0.054 (5)	0.330 (11)
H8A3	0.354750	0.708882	0.177077	0.064*	0.330 (11)
H8A4	0.408875	0.603398	0.167990	0.064*	0.330 (11)
C9A	0.48142 (19)	0.66155 (15)	0.23353 (7)	0.0454 (4)	
C10A	0.60548 (19)	0.76995 (15)	0.33746 (7)	0.0453 (4)	
C11A	0.6939 (2)	0.79817 (18)	0.37505 (8)	0.0580 (5)	
H11A	0.773514	0.764736	0.380089	0.070*	
C12A	0.6683 (2)	0.8734 (2)	0.40504 (9)	0.0664 (6)	
H12A	0.730490	0.891710	0.430239	0.080*	
C13A	0.5529 (2)	0.92254 (17)	0.39877 (9)	0.0594 (6)	
C14A	0.4648 (2)	0.89723 (17)	0.36131 (9)	0.0588 (5)	
H14A	0.385742	0.931387	0.356279	0.071*	
C15A	0.4919 (2)	0.82139 (17)	0.33077 (8)	0.0528 (5)	
H15A	0.430961	0.804786	0.304833	0.063*	
C16A	0.2677 (2)	0.58533 (16)	0.24393 (7)	0.0478 (5)	
C17A	0.2392 (2)	0.50222 (18)	0.21912 (10)	0.0633 (6)	
H17A	0.305254	0.456910	0.214024	0.076*	
C18A	0.1124 (3)	0.48517 (19)	0.20156 (10)	0.0689 (7)	
H18A	0.092331	0.427678	0.184344	0.083*	
C19A	0.0151 (2)	0.55004 (18)	0.20862 (8)	0.0574 (5)	
C20A	0.0455 (2)	0.63227 (18)	0.23423 (8)	0.0568 (5)	
H20A	−0.020731	0.677108	0.239908	0.068*	
C21A	0.1721 (2)	0.65056 (16)	0.25189 (7)	0.0521 (5)	
H21A	0.192371	0.707754	0.269352	0.062*	
C22A	0.6045 (2)	0.57437 (17)	0.37597 (8)	0.0578 (5)	
C23A	0.4110 (3)	1.0295 (3)	0.43679 (13)	0.0962 (10)	
H23A	0.378518	1.060752	0.406571	0.144*	
H23B	0.413342	1.075712	0.463558	0.144*	
H23C	0.353668	0.976341	0.444030	0.144*	
C24A	−0.1230 (3)	0.5333 (2)	0.18813 (11)	0.0803 (8)	
H24A	−0.183518	0.561316	0.210066	0.120*	
H24B	−0.139106	0.464541	0.185086	0.120*	
H24C	−0.135458	0.563409	0.156018	0.120*	
O1B	0.88611 (15)	0.55891 (11)	0.33264 (6)	0.0597 (4)	
O2B	1.17002 (16)	0.19933 (11)	0.42029 (6)	0.0623 (4)	
O3B	0.61297 (18)	0.20781 (13)	0.49274 (7)	0.0735 (5)	
N1B	1.05243 (16)	0.52114 (12)	0.38701 (6)	0.0442 (4)	
N2B	0.7586 (3)	0.37087 (19)	0.27435 (9)	0.0866 (8)	
C1B	0.96338 (19)	0.50067 (15)	0.34912 (7)	0.0445 (4)	
C2B	0.9722 (2)	0.40016 (16)	0.32852 (7)	0.0473 (5)	
H2B	1.045583	0.399447	0.306669	0.057*	
C3B	0.99990 (19)	0.32262 (14)	0.36769 (7)	0.0445 (4)	
H3B	1.028406	0.263711	0.350848	0.053*	
C4B	1.11285 (18)	0.35722 (14)	0.40066 (6)	0.0409 (4)	
C5B	1.19280 (19)	0.28396 (15)	0.42658 (7)	0.0459 (4)	
C6B	1.3012 (2)	0.31731 (17)	0.46160 (8)	0.0566 (5)	
H6BA	1.372469	0.270099	0.462105	0.068*	
H6BB	1.269539	0.320138	0.494725	0.068*	
C7B	1.3531 (2)	0.41394 (17)	0.44876 (8)	0.0546 (5)	
H7BA	1.398831	0.408795	0.418402	0.066*	
H7BB	1.416456	0.435436	0.475017	0.066*	
C8B	1.2457 (2)	0.48714 (16)	0.44186 (7)	0.0496 (5)	
H8BA	1.213782	0.503444	0.473927	0.060*	
H8BB	1.280653	0.546139	0.427828	0.060*	
C9B	1.13513 (18)	0.45094 (14)	0.40896 (6)	0.0409 (4)	
C10B	0.88665 (18)	0.29520 (14)	0.39738 (7)	0.0438 (4)	
C11B	0.8175 (2)	0.36257 (15)	0.42189 (8)	0.0490 (5)	
H11B	0.834148	0.428504	0.417023	0.059*	
C12B	0.7244 (2)	0.33612 (16)	0.45338 (8)	0.0529 (5)	
H12B	0.677947	0.383529	0.469731	0.064*	
C13B	0.6998 (2)	0.24062 (16)	0.46077 (8)	0.0524 (5)	
C14B	0.7626 (2)	0.17243 (16)	0.43470 (9)	0.0596 (6)	
H14B	0.742172	0.106745	0.438278	0.072*	
C15B	0.8550 (2)	0.19959 (16)	0.40346 (8)	0.0552 (5)	
H15B	0.897639	0.152078	0.385788	0.066*	
C16B	1.05339 (19)	0.61852 (14)	0.40490 (7)	0.0439 (4)	
C17B	1.0002 (2)	0.64075 (17)	0.44792 (8)	0.0591 (5)	
H17B	0.964595	0.591822	0.466862	0.071*	
C18B	0.9988 (3)	0.73479 (19)	0.46353 (9)	0.0659 (6)	
H18B	0.963120	0.749395	0.493561	0.079*	
C19B	1.0478 (2)	0.80763 (17)	0.43663 (9)	0.0593 (6)	
C20B	1.1019 (3)	0.78322 (18)	0.39393 (10)	0.0700 (7)	
H20B	1.137513	0.831935	0.374852	0.084*	
C21B	1.1056 (3)	0.68946 (17)	0.37820 (8)	0.0617 (6)	
H21B	1.144360	0.674336	0.348844	0.074*	
C22B	0.8523 (2)	0.38295 (17)	0.29799 (8)	0.0599 (6)	
C23B	0.5890 (3)	0.2675 (2)	0.53236 (9)	0.0676 (6)	
H23D	0.541756	0.324436	0.520484	0.101*	
H23E	0.671532	0.286706	0.549044	0.101*	
H23F	0.537152	0.232836	0.555109	0.101*	
C24B	1.0432 (3)	0.9097 (2)	0.45374 (13)	0.0892 (9)	
H24D	0.989531	0.913847	0.481671	0.134*	
H24E	1.006030	0.950037	0.427298	0.134*	
H24F	1.131171	0.931536	0.463385	0.134*	

### 4-(4-Methoxyphenyl)-1-(4-methylphenyl)-2,5-dioxo-1,2,5,6,7,8-hexahydroquinoline-3-carbonitrile (II) Atomic displacement parameters (Å^2^)

**Table:**

	*U*^11^	*U*^22^	*U*^33^	*U*^12^	*U*^13^	*U*^23^
O1A	0.0649 (10)	0.0884 (13)	0.0588 (9)	−0.0090 (9)	0.0108 (8)	0.0254 (9)
O2A	0.0504 (9)	0.0733 (11)	0.0676 (10)	−0.0076 (8)	0.0095 (7)	0.0054 (8)
O3A	0.0840 (13)	0.0771 (13)	0.0989 (14)	−0.0122 (10)	0.0082 (11)	−0.0350 (11)
N1A	0.0440 (9)	0.0576 (10)	0.0458 (9)	−0.0023 (7)	0.0052 (7)	0.0081 (7)
N2A	0.1003 (18)	0.0772 (15)	0.0541 (12)	0.0104 (13)	0.0028 (11)	0.0156 (10)
C1A	0.0551 (12)	0.0493 (11)	0.0472 (10)	0.0038 (9)	0.0099 (9)	0.0094 (9)
C2A	0.0532 (12)	0.0510 (12)	0.0457 (10)	0.0118 (9)	0.0045 (9)	0.0089 (9)
C3A	0.0378 (9)	0.0527 (11)	0.0482 (10)	0.0077 (8)	0.0025 (8)	0.0105 (9)
C4A	0.0429 (10)	0.0475 (11)	0.0425 (10)	0.0094 (8)	0.0077 (8)	0.0061 (8)
C5A	0.0464 (11)	0.0552 (12)	0.0522 (11)	0.0016 (9)	0.0115 (9)	0.0026 (9)
C6A	0.095 (5)	0.129 (6)	0.055 (4)	−0.062 (4)	−0.002 (3)	0.049 (4)
C7A	0.069 (3)	0.097 (4)	0.051 (2)	−0.014 (3)	−0.0021 (19)	0.029 (2)
C8A	0.058 (4)	0.069 (5)	0.046 (4)	−0.003 (3)	−0.009 (3)	0.002 (2)
C6AA	0.065 (7)	0.122 (12)	0.058 (8)	0.023 (7)	0.014 (6)	−0.023 (7)
C7AA	0.066 (5)	0.084 (7)	0.035 (3)	−0.013 (5)	0.008 (3)	0.006 (3)
C8AA	0.052 (8)	0.075 (9)	0.037 (7)	−0.018 (6)	0.024 (6)	0.010 (5)
C9A	0.0445 (10)	0.0503 (11)	0.0421 (10)	0.0058 (8)	0.0088 (8)	0.0049 (8)
C10A	0.0411 (10)	0.0502 (11)	0.0445 (10)	−0.0005 (8)	0.0026 (8)	0.0100 (8)
C11A	0.0454 (11)	0.0657 (14)	0.0616 (13)	0.0010 (10)	−0.0051 (10)	0.0044 (11)
C12A	0.0593 (14)	0.0718 (16)	0.0664 (14)	−0.0142 (12)	−0.0085 (11)	−0.0072 (12)
C13A	0.0596 (13)	0.0534 (13)	0.0660 (13)	−0.0135 (10)	0.0094 (11)	−0.0063 (10)
C14A	0.0508 (12)	0.0572 (13)	0.0686 (14)	0.0063 (10)	0.0046 (10)	0.0008 (11)
C15A	0.0466 (11)	0.0570 (12)	0.0538 (11)	0.0030 (9)	−0.0048 (9)	0.0006 (9)
C16A	0.0449 (10)	0.0540 (12)	0.0451 (10)	−0.0013 (9)	0.0082 (8)	0.0076 (9)
C17A	0.0542 (13)	0.0550 (13)	0.0811 (16)	0.0032 (10)	0.0071 (11)	−0.0048 (12)
C18A	0.0643 (15)	0.0568 (14)	0.0847 (17)	−0.0078 (11)	−0.0007 (13)	−0.0096 (12)
C19A	0.0530 (12)	0.0643 (14)	0.0552 (12)	−0.0051 (10)	0.0048 (10)	0.0117 (10)
C20A	0.0523 (12)	0.0664 (14)	0.0523 (11)	0.0096 (10)	0.0085 (9)	0.0076 (10)
C21A	0.0571 (12)	0.0535 (12)	0.0459 (10)	0.0008 (10)	0.0055 (9)	0.0016 (9)
C22A	0.0637 (14)	0.0543 (13)	0.0555 (13)	0.0103 (10)	0.0039 (10)	0.0096 (10)
C23A	0.090 (2)	0.094 (2)	0.106 (2)	0.0119 (18)	0.0168 (18)	−0.0366 (19)
C24A	0.0576 (15)	0.095 (2)	0.0873 (19)	−0.0087 (14)	−0.0062 (13)	0.0090 (16)
O1B	0.0548 (9)	0.0560 (9)	0.0661 (9)	0.0093 (7)	−0.0118 (7)	0.0051 (7)
O2B	0.0607 (9)	0.0479 (9)	0.0773 (10)	0.0092 (7)	−0.0046 (8)	0.0018 (7)
O3B	0.0720 (11)	0.0662 (11)	0.0845 (12)	−0.0222 (9)	0.0214 (9)	−0.0025 (9)
N1B	0.0465 (9)	0.0438 (9)	0.0420 (8)	0.0044 (7)	0.0004 (7)	−0.0016 (7)
N2B	0.0919 (17)	0.0799 (16)	0.0825 (15)	−0.0069 (13)	−0.0364 (14)	−0.0028 (12)
C1B	0.0426 (10)	0.0496 (11)	0.0412 (9)	0.0033 (8)	0.0011 (8)	0.0045 (8)
C2B	0.0483 (11)	0.0565 (12)	0.0367 (9)	0.0033 (9)	−0.0003 (8)	−0.0037 (8)
C3B	0.0472 (10)	0.0443 (10)	0.0418 (9)	0.0072 (8)	0.0007 (8)	−0.0078 (8)
C4B	0.0386 (9)	0.0482 (11)	0.0361 (9)	0.0049 (8)	0.0049 (7)	−0.0014 (8)
C5B	0.0437 (10)	0.0489 (12)	0.0453 (10)	0.0067 (8)	0.0062 (8)	−0.0010 (8)
C6B	0.0526 (12)	0.0586 (13)	0.0572 (12)	0.0100 (10)	−0.0076 (10)	0.0048 (10)
C7B	0.0434 (11)	0.0633 (14)	0.0558 (11)	0.0018 (9)	−0.0065 (9)	−0.0036 (10)
C8B	0.0485 (11)	0.0517 (12)	0.0478 (10)	0.0006 (9)	−0.0023 (9)	−0.0035 (9)
C9B	0.0402 (9)	0.0487 (11)	0.0342 (8)	0.0048 (8)	0.0051 (7)	−0.0017 (7)
C10B	0.0409 (9)	0.0455 (10)	0.0439 (9)	0.0006 (8)	−0.0058 (8)	−0.0040 (8)
C11B	0.0501 (11)	0.0409 (10)	0.0562 (11)	−0.0020 (8)	0.0052 (9)	−0.0017 (9)
C12B	0.0498 (11)	0.0520 (12)	0.0575 (12)	−0.0008 (9)	0.0070 (9)	−0.0061 (9)
C13B	0.0458 (11)	0.0551 (12)	0.0557 (11)	−0.0101 (9)	−0.0001 (9)	0.0018 (9)
C14B	0.0625 (13)	0.0432 (11)	0.0726 (14)	−0.0109 (10)	0.0000 (11)	−0.0026 (10)
C15B	0.0570 (13)	0.0439 (11)	0.0641 (13)	0.0013 (9)	−0.0010 (10)	−0.0111 (9)
C16B	0.0420 (10)	0.0467 (11)	0.0428 (9)	0.0054 (8)	0.0006 (8)	0.0003 (8)
C17B	0.0657 (14)	0.0561 (13)	0.0573 (12)	−0.0059 (11)	0.0186 (10)	−0.0036 (10)
C18B	0.0691 (15)	0.0678 (16)	0.0625 (14)	0.0010 (12)	0.0173 (12)	−0.0212 (12)
C19B	0.0551 (12)	0.0513 (13)	0.0697 (14)	0.0069 (10)	−0.0090 (11)	−0.0095 (11)
C20B	0.0912 (19)	0.0468 (13)	0.0725 (15)	−0.0048 (12)	0.0084 (14)	0.0094 (11)
C21B	0.0829 (16)	0.0534 (13)	0.0507 (12)	0.0017 (12)	0.0184 (11)	0.0030 (10)
C22B	0.0708 (15)	0.0574 (13)	0.0497 (11)	0.0034 (11)	−0.0101 (11)	−0.0021 (10)
C23B	0.0629 (14)	0.0806 (17)	0.0601 (13)	−0.0064 (13)	0.0093 (11)	0.0109 (12)
C24B	0.093 (2)	0.0580 (16)	0.114 (2)	0.0077 (15)	−0.0134 (18)	−0.0221 (16)

### 4-(4-Methoxyphenyl)-1-(4-methylphenyl)-2,5-dioxo-1,2,5,6,7,8-hexahydroquinoline-3-carbonitrile (II) Geometric parameters (Å, º)

**Table:**

O1A—C1A	1.204 (3)	C23A—H23B	0.9800
O2A—C5A	1.222 (3)	C23A—H23C	0.9800
O3A—C13A	1.369 (3)	C24A—H24A	0.9800
O3A—C23A	1.400 (4)	C24A—H24B	0.9800
N1A—C1A	1.380 (3)	C24A—H24C	0.9800
N1A—C9A	1.412 (3)	O1B—C1B	1.209 (2)
N1A—C16A	1.458 (3)	O2B—C5B	1.218 (3)
N2A—C22A	1.136 (3)	O3B—C13B	1.378 (3)
C1A—C2A	1.533 (3)	O3B—C23B	1.409 (3)
C2A—C22A	1.476 (3)	N1B—C1B	1.376 (3)
C2A—C3A	1.543 (3)	N1B—C9B	1.412 (3)
C2A—H2A	1.0000	N1B—C16B	1.449 (3)
C3A—C4A	1.510 (3)	N2B—C22B	1.145 (3)
C3A—C10A	1.516 (3)	C1B—C2B	1.522 (3)
C3A—H3A	1.0000	C2B—C22B	1.471 (3)
C4A—C9A	1.345 (3)	C2B—C3B	1.544 (3)
C4A—C5A	1.465 (3)	C2B—H2B	1.0000
C5A—C6A	1.487 (6)	C3B—C4B	1.511 (3)
C5A—C6AA	1.515 (13)	C3B—C10B	1.522 (3)
C6A—C7A	1.382 (7)	C3B—H3B	1.0000
C6A—H6A1	0.9900	C4B—C9B	1.349 (3)
C6A—H6A2	0.9900	C4B—C5B	1.472 (3)
C7A—C8A	1.490 (10)	C5B—C6B	1.504 (3)
C7A—H7A1	0.9900	C6B—C7B	1.506 (3)
C7A—H7A2	0.9900	C6B—H6BA	0.9900
C8A—C9A	1.503 (7)	C6B—H6BB	0.9900
C8A—H8A1	0.9900	C7B—C8B	1.514 (3)
C8A—H8A2	0.9900	C7B—H7BA	0.9900
C6AA—C7AA	1.481 (13)	C7B—H7BB	0.9900
C6AA—H6A3	0.9900	C8B—C9B	1.500 (3)
C6AA—H6A4	0.9900	C8B—H8BA	0.9900
C7AA—C8AA	1.556 (13)	C8B—H8BB	0.9900
C7AA—H7A3	0.9900	C10B—C11B	1.386 (3)
C7AA—H7A4	0.9900	C10B—C15B	1.391 (3)
C8AA—C9A	1.518 (14)	C11B—C12B	1.388 (3)
C8AA—H8A3	0.9900	C11B—H11B	0.9500
C8AA—H8A4	0.9900	C12B—C13B	1.378 (3)
C10A—C15A	1.380 (3)	C12B—H12B	0.9500
C10A—C11A	1.392 (3)	C13B—C14B	1.382 (3)
C11A—C12A	1.375 (4)	C14B—C15B	1.381 (3)
C11A—H11A	0.9500	C14B—H14B	0.9500
C12A—C13A	1.379 (4)	C15B—H15B	0.9500
C12A—H12A	0.9500	C16B—C21B	1.368 (3)
C13A—C14A	1.377 (3)	C16B—C17B	1.374 (3)
C14A—C15A	1.394 (3)	C17B—C18B	1.385 (3)
C14A—H14A	0.9500	C17B—H17B	0.9500
C15A—H15A	0.9500	C18B—C19B	1.376 (4)
C16A—C17A	1.371 (3)	C18B—H18B	0.9500
C16A—C21A	1.374 (3)	C19B—C20B	1.378 (4)
C17A—C18A	1.388 (4)	C19B—C24B	1.506 (3)
C17A—H17A	0.9500	C20B—C21B	1.383 (4)
C18A—C19A	1.380 (4)	C20B—H20B	0.9500
C18A—H18A	0.9500	C21B—H21B	0.9500
C19A—C20A	1.375 (4)	C23B—H23D	0.9800
C19A—C24A	1.519 (3)	C23B—H23E	0.9800
C20A—C21A	1.391 (3)	C23B—H23F	0.9800
C20A—H20A	0.9500	C24B—H24D	0.9800
C21A—H21A	0.9500	C24B—H24E	0.9800
C23A—H23A	0.9800	C24B—H24F	0.9800
			
C13A—O3A—C23A	119.0 (2)	O3A—C23A—H23B	109.5
C1A—N1A—C9A	122.95 (18)	H23A—C23A—H23B	109.5
C1A—N1A—C16A	116.59 (17)	O3A—C23A—H23C	109.5
C9A—N1A—C16A	120.30 (16)	H23A—C23A—H23C	109.5
O1A—C1A—N1A	122.7 (2)	H23B—C23A—H23C	109.5
O1A—C1A—C2A	123.00 (19)	C19A—C24A—H24A	109.5
N1A—C1A—C2A	114.25 (17)	C19A—C24A—H24B	109.5
C22A—C2A—C1A	109.44 (18)	H24A—C24A—H24B	109.5
C22A—C2A—C3A	112.57 (19)	C19A—C24A—H24C	109.5
C1A—C2A—C3A	113.61 (16)	H24A—C24A—H24C	109.5
C22A—C2A—H2A	106.9	H24B—C24A—H24C	109.5
C1A—C2A—H2A	106.9	C13B—O3B—C23B	116.91 (19)
C3A—C2A—H2A	106.9	C1B—N1B—C9B	122.42 (17)
C4A—C3A—C10A	113.21 (16)	C1B—N1B—C16B	116.28 (16)
C4A—C3A—C2A	107.67 (17)	C9B—N1B—C16B	121.24 (15)
C10A—C3A—C2A	114.14 (17)	O1B—C1B—N1B	122.5 (2)
C4A—C3A—H3A	107.1	O1B—C1B—C2B	122.54 (18)
C10A—C3A—H3A	107.1	N1B—C1B—C2B	114.93 (16)
C2A—C3A—H3A	107.1	C22B—C2B—C1B	107.18 (18)
C9A—C4A—C5A	120.91 (18)	C22B—C2B—C3B	113.26 (19)
C9A—C4A—C3A	122.37 (18)	C1B—C2B—C3B	113.74 (15)
C5A—C4A—C3A	116.70 (18)	C22B—C2B—H2B	107.5
O2A—C5A—C4A	121.5 (2)	C1B—C2B—H2B	107.5
O2A—C5A—C6A	122.6 (3)	C3B—C2B—H2B	107.5
C4A—C5A—C6A	116.0 (3)	C4B—C3B—C10B	110.53 (15)
O2A—C5A—C6AA	116.4 (6)	C4B—C3B—C2B	107.16 (17)
C4A—C5A—C6AA	122.0 (6)	C10B—C3B—C2B	116.00 (16)
C7A—C6A—C5A	119.8 (5)	C4B—C3B—H3B	107.6
C7A—C6A—H6A1	107.4	C10B—C3B—H3B	107.6
C5A—C6A—H6A1	107.4	C2B—C3B—H3B	107.6
C7A—C6A—H6A2	107.4	C9B—C4B—C5B	120.73 (17)
C5A—C6A—H6A2	107.4	C9B—C4B—C3B	122.01 (17)
H6A1—C6A—H6A2	106.9	C5B—C4B—C3B	117.10 (18)
C6A—C7A—C8A	114.0 (6)	O2B—C5B—C4B	120.75 (19)
C6A—C7A—H7A1	108.7	O2B—C5B—C6B	121.47 (19)
C8A—C7A—H7A1	108.7	C4B—C5B—C6B	117.77 (18)
C6A—C7A—H7A2	108.7	C5B—C6B—C7B	113.01 (18)
C8A—C7A—H7A2	108.7	C5B—C6B—H6BA	109.0
H7A1—C7A—H7A2	107.6	C7B—C6B—H6BA	109.0
C7A—C8A—C9A	113.4 (7)	C5B—C6B—H6BB	109.0
C7A—C8A—H8A1	108.9	C7B—C6B—H6BB	109.0
C9A—C8A—H8A1	108.9	H6BA—C6B—H6BB	107.8
C7A—C8A—H8A2	108.9	C6B—C7B—C8B	111.51 (18)
C9A—C8A—H8A2	108.9	C6B—C7B—H7BA	109.3
H8A1—C8A—H8A2	107.7	C8B—C7B—H7BA	109.3
C7AA—C6AA—C5A	110.6 (11)	C6B—C7B—H7BB	109.3
C7AA—C6AA—H6A3	109.5	C8B—C7B—H7BB	109.3
C5A—C6AA—H6A3	109.5	H7BA—C7B—H7BB	108.0
C7AA—C6AA—H6A4	109.5	C9B—C8B—C7B	111.81 (18)
C5A—C6AA—H6A4	109.5	C9B—C8B—H8BA	109.3
H6A3—C6AA—H6A4	108.1	C7B—C8B—H8BA	109.3
C6AA—C7AA—C8AA	123.9 (12)	C9B—C8B—H8BB	109.3
C6AA—C7AA—H7A3	106.4	C7B—C8B—H8BB	109.3
C8AA—C7AA—H7A3	106.4	H8BA—C8B—H8BB	107.9
C6AA—C7AA—H7A4	106.4	C4B—C9B—N1B	120.81 (17)
C8AA—C7AA—H7A4	106.4	C4B—C9B—C8B	123.09 (18)
H7A3—C7AA—H7A4	106.4	N1B—C9B—C8B	116.10 (18)
C9A—C8AA—C7AA	109.2 (12)	C11B—C10B—C15B	117.5 (2)
C9A—C8AA—H8A3	109.8	C11B—C10B—C3B	122.08 (18)
C7AA—C8AA—H8A3	109.8	C15B—C10B—C3B	120.23 (19)
C9A—C8AA—H8A4	109.8	C10B—C11B—C12B	121.7 (2)
C7AA—C8AA—H8A4	109.8	C10B—C11B—H11B	119.2
H8A3—C8AA—H8A4	108.3	C12B—C11B—H11B	119.2
C4A—C9A—N1A	120.92 (18)	C13B—C12B—C11B	119.6 (2)
C4A—C9A—C8A	121.5 (4)	C13B—C12B—H12B	120.2
N1A—C9A—C8A	117.5 (4)	C11B—C12B—H12B	120.2
C4A—C9A—C8AA	125.8 (7)	O3B—C13B—C12B	123.5 (2)
N1A—C9A—C8AA	113.1 (7)	O3B—C13B—C14B	116.8 (2)
C15A—C10A—C11A	117.6 (2)	C12B—C13B—C14B	119.7 (2)
C15A—C10A—C3A	123.09 (18)	C15B—C14B—C13B	120.1 (2)
C11A—C10A—C3A	119.35 (18)	C15B—C14B—H14B	119.9
C12A—C11A—C10A	121.4 (2)	C13B—C14B—H14B	119.9
C12A—C11A—H11A	119.3	C14B—C15B—C10B	121.2 (2)
C10A—C11A—H11A	119.3	C14B—C15B—H15B	119.4
C11A—C12A—C13A	120.4 (2)	C10B—C15B—H15B	119.4
C11A—C12A—H12A	119.8	C21B—C16B—C17B	119.6 (2)
C13A—C12A—H12A	119.8	C21B—C16B—N1B	119.62 (19)
O3A—C13A—C14A	125.0 (2)	C17B—C16B—N1B	120.78 (19)
O3A—C13A—C12A	115.6 (2)	C16B—C17B—C18B	119.7 (2)
C14A—C13A—C12A	119.4 (2)	C16B—C17B—H17B	120.1
C13A—C14A—C15A	119.9 (2)	C18B—C17B—H17B	120.1
C13A—C14A—H14A	120.1	C19B—C18B—C17B	121.7 (2)
C15A—C14A—H14A	120.1	C19B—C18B—H18B	119.2
C10A—C15A—C14A	121.4 (2)	C17B—C18B—H18B	119.2
C10A—C15A—H15A	119.3	C18B—C19B—C20B	117.4 (2)
C14A—C15A—H15A	119.3	C18B—C19B—C24B	120.9 (3)
C17A—C16A—C21A	120.6 (2)	C20B—C19B—C24B	121.7 (3)
C17A—C16A—N1A	120.6 (2)	C19B—C20B—C21B	121.6 (2)
C21A—C16A—N1A	118.7 (2)	C19B—C20B—H20B	119.2
C16A—C17A—C18A	119.1 (2)	C21B—C20B—H20B	119.2
C16A—C17A—H17A	120.4	C16B—C21B—C20B	120.0 (2)
C18A—C17A—H17A	120.4	C16B—C21B—H21B	120.0
C19A—C18A—C17A	121.3 (2)	C20B—C21B—H21B	120.0
C19A—C18A—H18A	119.3	N2B—C22B—C2B	179.0 (3)
C17A—C18A—H18A	119.3	O3B—C23B—H23D	109.5
C20A—C19A—C18A	118.6 (2)	O3B—C23B—H23E	109.5
C20A—C19A—C24A	119.8 (2)	H23D—C23B—H23E	109.5
C18A—C19A—C24A	121.6 (2)	O3B—C23B—H23F	109.5
C19A—C20A—C21A	120.8 (2)	H23D—C23B—H23F	109.5
C19A—C20A—H20A	119.6	H23E—C23B—H23F	109.5
C21A—C20A—H20A	119.6	C19B—C24B—H24D	109.5
C16A—C21A—C20A	119.5 (2)	C19B—C24B—H24E	109.5
C16A—C21A—H21A	120.2	H24D—C24B—H24E	109.5
C20A—C21A—H21A	120.2	C19B—C24B—H24F	109.5
N2A—C22A—C2A	178.0 (3)	H24D—C24B—H24F	109.5
O3A—C23A—H23A	109.5	H24E—C24B—H24F	109.5

### 4-(4-Methoxyphenyl)-1-(4-methylphenyl)-2,5-dioxo-1,2,5,6,7,8-hexahydroquinoline-3-carbonitrile (II) Hydrogen-bond geometry (Å, º)

*Cg*4 and *Cg*9 are the centroids of the C10*A*–C15*A* and C16*B*–C21*B* rings, respectively.

**Table:**

*D*—H···*A*	*D*—H	H···*A*	*D*···*A*	*D*—H···*A*
C21*A*—H21*A*···N2*B*^i^	0.95	2.65	3.258 (4)	123
C14*B*—H14*B*···O3*A*^ii^	0.95	2.65	3.417 (3)	139
C3*A*—H3*A*···O1*B*	1.00	2.37	3.171 (2)	136
C23*B*—H23*E*···*Cg*9^iii^	0.98	2.93	3.868 (3)	160
C23*B*—H23*F*···*Cg*4^iv^	0.98	2.74	3.710 (3)	169

Symmetry codes: (i) −*x*+1, *y*+1/2, −*z*+1/2; (ii) *x*, *y*−1, *z*; (iii) −*x*+2, −*y*+1, −*z*+1; (iv) −*x*+1, −*y*+1, −*z*+1.
